# Discrimination Index of Microcytic Anemia in Young Soldiers: A Single Institutional Analysis

**DOI:** 10.1371/journal.pone.0114061

**Published:** 2015-02-13

**Authors:** Tzu-Chuan Huang, Yi-Ying Wu, Yu-Guang Chen, Shiue-Wei Lai, Sheng-Cheng Wu, Ren-Hua Ye, Chieh-Sheng Lu, Jia-Hong Chen

**Affiliations:** 1 Division of Hematology/Oncology, Department of Medicine, Tri-Service General Hospital, Taipei, Taiwan; 2 Graduate Institute of Life Sciences, National Defense Medical Center, Taipei, Taiwan; 3 Graduate Institute of Clinical Medicine, College of Medicine, Taipei Medical University, Taipei, Taiwan; Northeastern University, UNITED STATES

## Abstract

**Background:**

The common differential diagnosis of microcytic anemia in young Asian men includes iron deficiency anemia (IDA), α-thalassemia (αT) and β-thalassemia (βT). In this study, we aimed to distinguish between these diseases in a distinct population of young men using a specific index.

**Patients and Methods:**

We retrospectively reviewed the laboratory data of young men with microcytic anemia. The clinical, characteristic and laboratory data, including complete blood cell counts, serum ferritin and hemoglobin electrophoresis results, were collected; genomic DNA mutations were also evaluated. Based on these data, ten discrimination indices were used to differentiate thalassemia from IDA. The sensitivity, specificity, positive and negative predictive values, Youden’s index and receiver operating characteristic (ROC) curves were also calculated.

**Results:**

A total of 877 patients (92 patients with IDA, 332 with αT and 453 with βT) were enrolled; the Shine and Lal (S&L) formula was the best method with which to discriminate IDA from thalassemia (100% sensitivity, 91% specificity). The new cut-off values were evaluated, and the approaches used in our study cohort, particularly the Green & King (G&K) formula, significantly increased the accuracies of red cell distribution width-containing indices (cut-off value: 58.66; 89.62% sensitivity and 96.2% specificity; AUC: 0.9716). In addition, when applied properly, these indices could differentiate IDA patients from αT patients, especially Huber-Herklotz index (HH).

**Conclusions:**

The sensitivity and specificity differed among ethnic and age groups. We concluded that when using the original cut-off value, the S&L formula was the best discriminating index for differentiating between IDA and thalassemia in young Asian men. However, when using the G&K formula, the newly obtained cut-off value must be applied to increase accuracy based on the results from our cohort.

## Introduction

Microcytic anemia is most commonly caused by iron deficiency anemia (IDA) and thalassemia. IDA is a worldwide nutrition-related disease that is more prevalent in women than in men (9–20% and 2%, respectively) [1]. IDA may result from insufficient iron intake, menstrual bleeding in women of childbearing age, or chronic blood loss in the gastrointestinal tract [2]. Thalassemia is an inherited hemoglobin pathology that results from defective synthesis of the gene encoding the globin chain of adult hemoglobin A [3]. This condition can be classified as α-thalassemia (αT) or β-thalassemia (βT), depending on which globin carries the mutation. Thalassemia patients produce abnormal red blood cells that are easily destroyed and thus significantly reduce the hemoglobin (Hb) levels and the mean corpuscular volume (MCV). The global prevalence of thalassemia is approximately 5%, although this varies among different populations with increased frequencies in the Mediterranean region, Africa and Asia. The clinical presentation of thalassemia ranges from nearly asymptomatic to severe anemia requiring lifelong blood transfusions, which can cause complications in multiple organ systems. The gold standard of therapy for symptomatic thalassemia is red blood cell transfusion, which then necessitates iron chelation therapy. Silent carriers of alpha thalassemia and persons with either alpha or beta thalassemic traits are asymptomatic and require no treatment [4]. Nowadays, the diagnosis of IDA depends on the serum ferritin level and transferrin saturation level instead of iron staining in bone marrow biopsies. However, the diagnosis of thalassemia depends on hemoglobin electrophoresis, high-performance liquid chromatography and DNA testing, all of which require significant expenditures of time and resources to confirm the diagnosis. It is therefore clinically important to have a simple index that can effectively differentiate between IDA and thalassemia.

Several discriminating indices proposed in previous publications were designed to differentiate between IDA and βT. These indices include the Mentzer Index (MI) [5], Green & King Index (G&K) [6], Red Cell Distribution Width Index (RDWI) [7], and England & Fraser Index (E&F) [8], as well as several others. Red blood cell (RBC) count, Hb, MCV and RDW are usually integrated in these mathematical formulas. For example, MI (MCV/RBC) can easily help to distinguish between IDA and thalassemia for children [5]. The ratio is usually less than 13 in thalassemia whereas iron deficiency yields values greater than 13. However, the sensitivities and specificities of these indices varied, and no single perfect index exists that is suitable for every person. Because these indices were developed in the contexts of different populations, generations and genders, they may be neither suitable nor easily applied to daily clinical use. Previous studies have primarily focused on distinguishing IDA from βT groups without regard to patients with αT. Therefore, few effective screening indices were introduced to identify αT patients, except Huber-Herklotz index (HH) [9]. HH values less than 20 are almost exclusively encountered in αT. On the other hand, IDA is strongly suggested with HH values more than 23. Batebi et al. [10] demonstrated that the results of these indices appeared to be more reliable for men than for women, a finding that is related to the higher prevalence of IDA among women than men. In this study, we focused on a population of young Taiwanese men with microcytic anemia to examine the sensitivity and specificity of each index. We also evaluated whether another cut-off value might be more suitable for this subpopulation to improve the efficacy of clinical differentiation between these diseases.

## Materials and Methods

### Patients and study design

We retrospectively reviewed the laboratory data of young male soldiers with microcytic anemia (MCV <80 and Hb <14 g/dL) at the Tri-Service General Hospital between 2009 and 2012. Patients with evidence of inflammatory disorders, malignancy, anemia consequent to chronic disease or a history of acute hemorrhage were excluded from this study. The laboratory data, including complete blood cell counts, serum ferritin levels, and hemoglobin electrophoresis results, were recorded. Genomic DNA was extracted from the peripheral blood via standard methods to confirm diagnosis. Multiplex polymerase chain reaction (PCR) analyses were employed to confirm the three subtypes of α-thalassemia minor (α-TM): southeast Asian deletion (—SEA), Thailand deletion (—THAI), and Philippine deletion (—FIL) [11]. We retrieved these results retrospectively via a chart review. Diagnosis of the β-thalassemia trait (β-TT) was based on the presence of hypochromia and microcytosis on the peripheral blood smear, normal ferritin levels and a HbA2 >3.5%, whereas low serum ferritin levels (<22 ng/ml) were considered to indicate a diagnosis of IDA. Additionally, ten discrimination indices were evaluated and compared. This study was conducted under the guidelines of the Helsinki Declaration and approved by the Human Subjects Protection Offices (IRB) at the Tri-Service General Hospital. Because all identifying patient information was removed prior to analysis in this study, informed consent was not obtained.

The ten discrimination indices that were examined were as follows:

Mentzer Index (MI) [5]: MCV/RBC
England & Fraser Index (E&F) [8]: MCV − RBC- (5 × Hb) − k [k = 6.4]
Shine and Lal formula (S&L) [12]: MCV^2^ × MCH/100
Ehsani formula (EF) [13]: MCV—10 × RBC
Srivastava formula (SF) [14]: MCH/RBC
Palestinian population (PP) [15]: MCV—RBC—3 × Hb
Green & King Index (G&K) [6]: MCV^2^ × RDW /(Hb × 100)
RDW Index (RDWI) [7]: MCV × RDW/RBC
(R) [7]: RDW/RBC
Huber-Herklotz Index (HH) [9]: (MCH × RDW × 0.1/RBC)+ RDW

### Statistical analysis

The data were analyzed using SPSS software, version 19 (SPSS Inc., Chicago, IL, USA). One-way ANOVA was used to compare parameters among the patients diagnosed with IDA, α-TM or β-TT. The sensitivity and specificity of each index was calculated according to the previous cut-off value. Receiver operating characteristic (ROC) curves were plotted to calculate the areas under the curve (AUC) to determine the proper cut-off values in our patient population. The sensitivity, specificity, positive and negative predictive values, efficacy, and Youden’s index (YI) were calculated for each index and formula.

## Results

A total of 877 draftees were enrolled, of whom 92 subjects were confirmed to have IDA, 332 to have α-TM and 453 to have β-TT based on their respective ferritin level, Hb electrophoresis, and genomic PCR testing results. The baseline characteristics and laboratory data are summarized in Table 1. Significant differences (*p*<0.0001) were observed among the three groups in all parameters except age (*p* = 0.061). The most common gene abnormality in the α-TM patients in our analysis was—SEA (95.7%), followed by—THAI (3.3%) and—FIL (0.9%). The accuracies of the individual mathematical formulas and indices are shown in Tables 2 and 3 according to previously published standard cut-off values. Table 2 shows that S&L was the most reliable for differentiating all thalassemias from IDA, with 100% sensitivity and 91% specificity. Additionally, formulas containing the RDW parameter, including G&K, RDWI, R and HH, yielded YI values greater than 0.7 as well as acceptable sensitivities and specificities. Nevertheless, reduced sensitivity (67%) was observed when using MI in our cohort, although this is considered to be a common and simple parameter for distinguishing thalassemia from IDA in clinical practice. The ten indices/formulas that were applied here can be properly used in α-TM patients (Table 3). There was no doubt that HH owned the best accuracy with YI of 0.84, followed by S&L (0.83) for differentiating α-TM from IDA. Compared with α-TM patients, the discernment of HH used in β-TT patients is relatively poorer with reduced sensitivity (77%), specificity (97%) and YI (0.75). However, S&L showed the opposite results with slightly better sensitivity (100%), specificity (86%) and YI (0.86). The applications of MI, EF, SF and PP appeared to provide less accuracy in α-TM patients (YI < 0.7). All ten discrimination indices were unable to consistently and correctly identify patients with either α-TM or β-TT (data not shown).

**Table 1:** Baseline characteristics, hematological parameters and results of different discrimination index among the three study groups.

	IDA	Alpha	Beta	*p* value
Number	92	332	453	
Age	21.6 ± 2.1 (18–29)	22.3 ± 2.6 (18–32)	22.2 ± 2.2 (18–32)	0.061
RBC	5.37 ± 0.61 (3.25–6.74)	5.94 ± 0.37 (4.57–7.02)	6.14 ± 0.46 (3.88–7.31)	<0.0001
Hb	11.1 ± 1.7 (6.3–13.5)	12.6 ± 0.7 (8.6–13.9)	12.2 ± 0.8 (8.0–13.9)	<0.0001
MCV	69.0 ± 6.4 (51.0–79.4)	67.4 ± 3.3 (51.2–76.9)	62.4 ± 3.4 (50.3–77.2)	<0.0001
MCH	20.9 ± 3.0 (14.0–28.9)	21.3 ± 1.3 (10.8–24.1)	20.0 ± 1.1 (15.9–25.3)	<0.0001
PLT	301.6 ± 78.1 (157–580)	236.4 ± 55.3 (93–545)	237.6 ± 49.1 (86–407)	<0.0001
RDW	17.7 ± 2.1 (11–23.4)	15.2 ± 1.6 (12.6–25.2)	15.6 ± 1.1 (13.4–21.6)	<0.0001
Ferritin	5.4 ± 3.5 (0.5–21.6)	168.8 ± 108.7 (22.8–987.8)	221.5 ± 114.3 (24.6–836.2)	<0.0001
HbA+F	97.9 ± 0.3 (97.3–98.9)	97.7 ± 0.3 (96.4–99.8)	94.5 ± 0.4 (92.5–96.1)	<0.0001
HbA2	2.1 ± 0.3 (1.1–2.7)	2.3 ± 0.3 (0.2–3.0)	5.5 ± 0.4 (3.9–7.5)	<0.0001
MI	13.1 ± 2.4 (7.57–24.03)	11.4 ± 1.1 (7.50–14.68)	11.0 ± 1.3 (7.34–18.14)	<0.0001
E&F	1.5 ± 7.1 (-15.26–21.41)	-8.0 ± 4.5 (-19.74–11.23)	-11.4 ± 5.4 (-24.44–17.72)	<0.0001
S&L	1025 ± 299 (385–1545)	973 ± 140 (412–1356)	783 ± 131 (402–1508)	<0.0001
EF	15.3 ± 10.3 (-16.4–45.6)	8.1 ± 6.2 (-17.1–23.9)	0.9 ± 7.2 (-18.9–33.7)	<0.0001
SF	4.0 ± 0.8 (2.2–7.2)	3.6 ± 0.4 (1.8–4.7)	3.3 ± 0.4 (2.3–5.9)	<0.0001
PP	30.2 ± 5.6 (14.3–43.3)	23.6 ± 3.7 (11.8–34.8)	19.5 ± 4.4 (8.5–40.7)	<0.0001
G&K	78.0 ± 15.6 (50.2–141.67)	54.6 ± 6.8 (42.4–124.6)	49.6 ± 7.9 (38.7–124.9)	<0.0001
RDWI	229.7 ± 41.4 (161.5–371.9)	172.0 ± 19.6 (136.4–359.5)	158.9 ± 23.9 (121.3–378.6)	<0.0001
R	3.3 ± 0.6 (2.5–5.9)	2.6 ± 0.3 (2.1–5.5)	2.6 ± 0.3 (2.1–5.6)	<0.0001
HH	24.5 ± 2.6 (23.9–25.1)	20.6 ± 1.7 (20.4–20.8)	20.8 ± 1.5 (20.6–20.9)	<0.0001

Abbreviations: Age, years; RBC: red blood cell count, 10^12/L; Hb: hemoglobin, g/dL; MCV, fL; MCH, pg; PLT: platelet count, 10^6/L; RDW, %; Ferritin, ng/mL; HbA+F, %; HbA2, %.

**Table 2:** The evaluation of different discrimination index in differentiation of thalassemia from IDA according to the previously published standard cut-off values.

	Cut-off	PPV (%)	NPV (%)	EFF (%)	Sensitivity (%)	Specificity(%)	Youden’s index
MI	<13	94	54	74	67	90	0.58
E&F	<0	96	66	81	74	94	0.68
S&L	<1530	90	100	95	100	91	0.91
EF	<15	94	44	69	63	89	0.52
SF	<3.8	95	29	62	57	84	0.41
PP	<27	97	46	71	64	93	0.57
G&K	<72	95	80	87	83	94	0.77
RDWI	<220	94	77	86	81	93	0.74
R	<3.3	94	73	83	77	92	0.70
HH	<21/>23	99	63	81	73	99	0.71

Abbreviations: PPV: positive predictive value, NPV: negative predictive value, EEF: efficiency

**Table 3:** The evaluation of different discrimination index in differentiation of α-thalassemia minor from IDA according to the previously published standard cut-off values.

	Cut-off	PPV (%)	NPV (%)	EFF (%)	Sensitivity (%)	Specificity (%)	Youden’s index
MI	<13	87	65	76	71	83	0.54
E&F	<0	90	82	86	83	89	0.73
S&L	<1530	79	100	89	100	83	0.83
EF	<15	87	53	70	65	80	0.46
SF	<3.8	86	37	62	58	73	0.31
PP	<27	90	55	71	63	87	0.50
G&K	<72	88	92	90	91	89	0.80
RDWI	<220	88	89	88	89	88	0.77
R	<3.3	86	84	95	84	86	0.70
HH	<21/>23	98	84	91	86	98	0.84

Abbreviations: PPV: positive predictive value, NPV: negative predictive value, EEF: efficiency

The best cut-off values for our cohort were calculated according to the AUC results, and the corresponding sensitivities and specificities of each formula are summarized in Tables 4 and 5. Among the ten indices, the G&K formula appeared to be the best predictor for identifying all of the thalassemia groups (Table 4). In addition to an increase in sensitivity from 83.6% to 89.62%, the specificity also increased from 94% to 96.2% when the cut-off value of the G&K formula was adjusted to less than 58.66 (AUC = 0.9716; *p*<0.0001). The same result was observed with the RDWI formula. However, markedly decreased sensitivity and specificity were observed when the cut-off value of S&L was adjusted to less than 948.5. Seven of the ten formulas and indices (excluding S&L, G&K and RDWI) exhibited increased sensitivity but impaired specificity when we applied the new cut-off points in our analysis. The same results were observed in a subgroup analysis of α-TM patients (Table 5).

**Table 4:** The proposed cut-off values of different discrimination index in differentiation of thalassemia from IDA.

	Cut-off	Sensitivity (%)	Specificity (%)	Likelihood ratio	95% CI	AUC	*p* value
MI	<12.05	85.28	66.67	2.56	0.7585–0.8665	0.8125	<0.0001
E&F	<-6.475	77.72	88.89	6.99	0.8778–0.9477	0.9128	<0.0001
S&L	<948.5	70.04	61.11	1.80	0.5830–0.7400	0.6615	<0.0001
EF	<12.45	87.32	63.33	2.38	0.7552–0.8635	0.8093	<0.0001
SF	<3.935	89.63	53.33	1.92	0.6236–0.7720	0.6978	<0.0001
PP	<25.45	82.07	83.33	4.92	0.8567–0.9342	0.8955	<0.0001
G&K	<58.66	89.62	96.20	23.6	0.9551–0.9882	0.9716	<0.0001
RDWI	<177.1	81.37	94.94	16.07	0.9393–0.9783	0.9588	<0.0001
R	<2.805	90.38	87.34	7.14	0.9155–0.9634	0.9395	<0.0001
HH	<22	89.91	88.61	7.89	0.8665–0.9602	0.9234	<0.0001

**Table 5:** The proposed cut-off values of different discrimination index in differentiation of α-thalassemia minor from IDA.

	Cut-off	Sensitivity (%)	Specificity (%)	Likelihood ratio	95% CI	AUC	*p* value
MI	<12.75	88.18	55.56	1.98	0.6593–0.8024	0.7309	<0.0001
E&F	<-4.230	83.03	85.56	5.75	0.8393–0.9322	0.8857	<0.0001
S&L	<1082	83.03	48.89	1.62	0.4744–0.6506	0.5625	<0.0001
EF	<18.00	96.06	47.78	1.84	0.6509–0.7950	0.7229	<0.0001
SF	<4.080	91.82	45.56	1.69	0.5431–0.7120	0.6275	0.0002
PP	<26.13	78.48	81.11	4.16	0.7920–0.8997	0.8459	<0.0001
G&K	<64.46	94.49	91.14	10.66	0.9388–0.9889	0.9639	<0.0001
RDWI	<191.9	89.34	91.14	10.08	0.9153–0.9742	0.9447	<0.0001
R	<2.845	90.81	84.81	5.98	0.9091–0.9635	0.9363	<0.0001
HH	<22	89.67	88.61	7.87	0.8872–0.9640	0.9256	<0.0001

## Discussion

Differentiation between the two different types of microcytic anemia related to thalassemia and IDA is clinically significant. Particularly in Southeast Asia, thalassemia is a public health issue that requires the use of an accurate discrimination index before performing hemoglobin electrophoresis and DNA analyses. A good predictive index could quickly screen for the disease and increase the cost effectiveness of treatments. Since 1973, several indices such as G&K, E&F, MI, RDWI, RBC count, and the RDW have been introduced in an attempt to inexpensively and simply distinguish between IDA and thalassemia as well as prevent any adverse effects from long-term iron therapy. However, none of these indices has been considered optimal as their sensitivities and specificities are less than 100%, a feature primarily due to differences in race, study population (mostly βT and IDA) and study region. As the prevalence of αT is higher in Southeast Asia, it is important to validate the accuracies of these indices for differentiating between α-TM and IDA. Our study aimed to determine which index or formula could best screen for thalassemia correctly and rapidly in young Taiwanese male patients with microcytic anemia as well as to prove that these indices could be used in α-TM patients. In our cohort, the S&L formula worked well and achieved 100% sensitivity and 91% specificity when using its original cut-off value of less than 1530, a result similar to that reported by Rathod et al. in an Indian population [16]. It must be noted that the S&L formula had the highest false positive rate (10%) relative to the other 9 indices. The most likely reason for this difference might be related to its higher original cut-off value. MI is another common and practical predictive index; however, we observed a lower sensitivity (67%) with this index in comparison to previous reports (82–95%) [13,15–17]. Significantly higher mean RDW values and red blood cell anisocytosis are known observations in iron-deficient patients, and these findings can increase the discrimination accuracy between thalassemia and IDA [18]. Table 6 summarizes different study results that focused on the RBC discriminating index in thalassemia patients (who were nearly all βT) and normal individuals of different ages and genders in various countries. This approach tended to favor RDW-containing regimens such as RDWI and G&K. However, MI and S&L were also suggested by other studies. It is concerning that the cut-off values differed according to ethnicity, gender, and age. We used ROC curves to evaluate each index and define more accurate cut-off values for our study and found that G&K and other RDW-containing indices worked well in our young Asian male population after adjusting the new cut-off values.

**Table 6:** summarizes different study results that focused on the RBC discrimination index in thalassemia patients and normal individuals of different ages and genders in various countries.

Studies	Ethnic group	Case	Number	Youden Index	Favor index
		αT	βT		
Janel et al. [24]	France		49	0.83	G&K
				0.83	RDWI
Demir et al. [25]	Turkey		37	0.8	RDWI
Shen et al. [26]	China		127 (children)	0.81	RDWI
Matos et al. [27]	Brazil		47	0.75	RDWI
Ferrara et al. [28]	Italy		215 (children)	0.64	E&F
Nalbantoğlu et al. [29]	Turkey		62 (children)	0.58	E&F
Nesa et al. [30]	Bangladesh		57	N/A	RDWI
Ntaios et al. [31]	Greece		373	0.71	G&K
Beyan et al. [32]	Turkey		66	0.74	RBC
Okan et al. [33]	Turkey		100	0.91	S&L
Ehsani et al. [13]	Iran		154	0.9	MI
Urrechaga [2]	Spain		150	0.69	MI
Sirdah et al. [15]	Palestine		1272	0.69	G&K
				0.68	RDWI
Batebi et al. [10]	N/A		273 (male)	0.88	MI
Mussarrat et al. [34]	IRAN		223	0.72	RDWI
AlFadhli et al. [35]	Kuwait.	50	47	0.98	E&F
Our study	Taiwan	332	453 (male)	0.91	S&L

The reported prevalence rates of α-TM and β-TT in Taiwan were 3.4% [19] and 1.1% [20], respectively. Given the lack of effective screening tests, DNA analysis remains the current gold standard for the accurate diagnosis of αT. Pranpanus et al. [21] reported that the mean corpuscular hemoglobin (MCH) levels are a better parameter for screening α-TM and β-TT at a cut-off value of less than 26.5. However, Mehdi and Al Dahmash [22] published a conflicting result; specifically, when the same RBC indices were used, the MCV and RBC counts failed to distinguish between αT and βT carriers. In our study, we presented the largest cohort of α-TM patients and found that the RBC discriminating index could be properly applied to distinguish not only β-TT but also α-TM from IDA. Because HH specifically demonstrated the best accuracy to differentiate α-TM from IDA in our study, it is an important evidence to support the clinical application to screen α-TM. In addition, HH and S&L indices both contained MCH, which may be a clue to improve other RBC indices and to establish new formulas when facing the problem to distinguish between α-TM and IDA. However, none of the indices can easily distinguish between α-TM and β-TT. Further testing involving the RBC indices will be necessary to better identify patients with α-TM [23].

In conclusion, our study demonstrated that the S&L formula was the best index for distinguishing thalassemia from IDA in young Asian men with microcytic anemia. HH is more accurate and specific to identify α-TM from IDA. Using our adjusted cut-off values, we observed that the G&K index and RDWI exhibited improved sensitivity and specificity in IDA patients relative to either α-TM or β-TT patients. However, none of the known indices could differentiate between α-TM and β-TT, indicating the possible need for a new index as well as other tests to better distinguish these two thalassemia subtypes.
